# Predictors of health state utility values using SF-6D for Chinese adult patients with β-thalassemia major

**DOI:** 10.3389/fpubh.2022.1072866

**Published:** 2023-01-19

**Authors:** Runqi Zhang, Shuo Zhang, Jing Ming, Jing Xie, Baoguo Liu, Cuiqian Chen, Xiaojie Sun, Xuemei Zhen

**Affiliations:** ^1^Centre for Health Management and Policy Research, School of Public Health, Cheeloo College of Medicine, Shandong University, Jinan, Shandong, China; ^2^National Health Commission (NHC) Key Lab of Health Economics and Policy Research, Shandong University, Jinan, Shandong, China; ^3^Beijing New Sunshine Charity Foundation, Beijing, China

**Keywords:** health utility, β-thalassemia, adult, quality of life, SF-6D

## Abstract

**Background:**

Patients with β-thalassemia major (β-TM), predominantly adult patients, are associated with physical, mental, and social problems, that result in decreased quality of life (QoL). However, there is a paucity of data on QoL and health status utility (HSU) among adult patients with β-TM in mainland China. Our study aimed to evaluate the QoL by short form 36 questionnaire (SF-36) of adult patients with β-TM in mainland China and to estimate their HSU by SF-6D. In addition, we aimed to identify predictors of HSU.

**Methods:**

In this cross-sectional descriptive study, a total of 75 adult patients with β-TM were included by the snowball sampling method that applied involving seven provinces with a relatively high prevalence of thalassemia across mainland China between September 1, 2021 and January 31, 2022. The collected information included social-demographic characteristics, health conditions, treatment, social support (social support rating scale), caregiver burden (Zarit burden interview), and QoL (SF-36). HSU scores were calculated for each adult patient from their SF-36 responses using the SF-6D algorithm with Hong Kong's tariff. The frequency of participants' responses to the SF-6D for each item of the options was described. Mean HSU scores between different subgroups were calculated. Ordinary least squares (OLS) regression modeling was performed to identify factors associated with HSU.

**Results:**

A total of 75 adult patients with β-TM were included in this study. The mean SF-36 score was 50.2 ± 10.70, of which physical and mental scores were 47.57 ± 11.28 and 52.85 ± 14.21, respectively. In addition, the mean SF-6D utility score was estimated to be 0.598 ± 0.112, ranging from 0.391 to 0.962. Univariate analyses showed that interruption of iron chelation treatment significantly affected HSU values (*P* = 0.038); diagnosis with comorbidity very slightly affected HSU values (*P* = 0.0996). In the multivariate analysis, diagnosis with comorbidity (*P* = 0.042) was significantly negatively associated with HSU values; the minimum pre-transfusion hemoglobin concentration (*P* = 0.047) and social support (*P* = 0.068) were positively associated with HSU values.

**Conclusion:**

This study presents poor QoL and HSU outcomes in Chinese adult patients with β-TM. The study also highlights the importance of social support and treatment compliance, which can increase hemoglobin content and reduce comorbidities, further to ensure the QoL of patients. These findings can be used for future clinical and economic studies.

## 1. Introduction

Thalassemia, as a regional and global problem, is widespread in Southeast Asia, the Mediterranean, and southern China (1). About 1.5% of the global population (about 80–90 million people) were heterozygotes or carriers of β-thalassemia, and ~26,000 patients with β-thalassemia major (β-TM) were born yearly (2). Globally, the annual prevalence of symptomatic patients with β-thalassemia is ~1 in 100,000, up to 90% of these births occur in low- or middle-income countries (2). In China, an epidemiological study with a population of nearly 600,000 revealed that the prevalence of β-thalassemia was 0.67% (3), and the overall prevalence in high-incidence provinces was roughly 2.21% (4); however, there was still lacking accurately regional epidemiological distribution.

β-TM, as the most severe form, required regular blood transfusions and iron chelation therapy throughout life (5). Lifetime transfusions would lead to serious iron overload, which led to organ damage, dysfunction, complications, and eventually death (1). It was reported that 89% of patients with β-TM lived beyond 40 years in Greece, and 80% of patients lived beyond 45 years in the United Kingdom (6), while patients between 2011 and 2017 in Guangxi province, China, could just live to 28 years averagely (7). With recent advances in treatment technology in China, the overall life expectancy of patients seems to have improved, but it remains to be revealed through further epidemiological data. But the extension of life expectancy has led to an increase in the prevalence of co-morbidities, resulting in a serious deterioration of quality of life (QoL) of patients (8).

The healthcare cost for patients with β-TM is proportional to their age and weight, and long-term treatment imposes a prohibitive financial burden of the disease on individuals and families (9). Hematopoietic stem cell transplantation offers a cure for thalassemia, but the optimal age for the procedure has been missed in adult patients (10). The amount and frequency of blood transfusions and iron chelation increase with age and weight, and so did the direct medical costs (11). Meanwhile, limitations in physical function and mobility prevented adult patients from managing their daily lives and undertaking normal tasks. The short transfusion cycle resulting in frequent outpatient clinics and hospitalization took up a lot of time, and adult patients had to choose some casual jobs with less stringent requirements on working hours which even were rejected by many employers, adult patients had difficulty in obtaining stable employment (12). They faced more challenges from work and employment which further aggravated the financial strain on patients and their families. It was estimated that the lifetime medical burden for patients in the UK was £803,000 (13), the annual financial burden for patients in Iran was $8,321.8 (11), the mean 1-year healthcare cost for patients in the US was $128,062 (14), and the annual direct financial burden of disease for patients in Guangdong Province, China was CNY 43,058.7 (15).

The WHO (16) defined the QoL as: “An individual's perception of their position in life in the context of the culture and value systems in which they live and concerning their goals, expectations, standards, and concerns” (17). Patients with β-TM had a substantially lower QoL in comparison with the general population (18). Many studies have shown that the diagnosis of β-TM negatively impacted the QoL of patients (19, 20). However, most of studies have been limited to the pediatric and adolescent β-TM population. With aging, adult patients have a worse disease burden and financial burden, and a significantly lower QoL than pediatric patients (21). Moreover, the treatment options for adult patients were limited, and the government and society lacked targeted efforts to help and support them. Thus, the suboptimal survival environment and condition of adult patients with β-TM seriously affects their QoL and warrants further study.

In addition, the identification of HSU and its predictors, especially modifiable ones such as social support and caregiver burden, is of practical significance to further improve the QoL with β-TM in China and to continuously increase their life expectancy. As for the social support, Shumaker and Brownell (22) defined it as “an exchange of resources between two individuals perceived by the provider or the recipient to be intended to enhance the wellbeing of the recipient” (23). Thalassemia patients need more social support than the general population (17). One study pointed out that improving social support for thalassemia patients and raising public awareness about the incidence and treatment of thalassemia could be useful to improve the condition of patients and their QoL (23). Caregiver burden has been defined by Zarit (24) as the attitudes and emotional-affective reactions from the experiences of the role with repercussions in the personal, family, and social spheres (25). Parents of thalassemia patients suffer higher levels of distress compared to others (26). The caregiver burden affects the adherence of thalassemia patients and their families to the treatment of the disease, which in turn impacts the QoL and the ability of patients to integrate into society, and ultimately the health of the patients (27).

To our knowledge, based on the existing literature, numerous studies have been conducted worldwide to assess QoL in β-TM patients (18), while rarely in terms of health status utility (HSU). In addition, almost all the recently published analyses on the QoL in β-TM patients were from middle eastern countries, mainly focusing on children (19, 20). There has been no study describing HSU values and its predictors for adult patients with β-TM in mainland China.

Therefore, we aimed to investigate the QoL of adult patients with β-TM using the SF-36 in mainland China, to measure HSU by the SF-6D algorithm with Hong Kong population norms based on the response of each patient, to compare mean utility scores for subgroups of adult patients with β-TM, and to develop a multivariable regression model to identify the factors associated with the HSU values.

## 2. Materials and methods

### 2.1. Study design

This was a cross-sectional descriptive study. Considering the lack of epidemiological parameters nationwide, as well as the extremely small number of adult patients with scattered residence, and the great difficulty and huge costs to implement the investigation during COVID-19. An online survey with the “questionnaire star (https://www.wjx.cn)” was conducted using snowball sampling at a national level between September 1, 2021, and January 31, 2022, including seven provinces with a relatively high prevalence of thalassemia namely Guangdong Province and Guangxi Zhuang Autonomous Region, Fujian Province, Jiangsu Province, Jiangxi Province, Hunan Province, and Xinjiang Uygur Autonomous Region. On the one hand, patient recruitment information was reprinted and forwarded through the website of Beijing New Sunshine Charity Foundation and the Thalassemia Mutual Aid WeChat groups nationally. On the other hand, physician referral from medical institutions was another important channel as well.

### 2.2. Inclusion and exclusion criteria

The study sample consisted of 75 adult patients who were genetically diagnosed as β-TM, and a primary caregiver of each patient participated in the study (*N* = 75). Patients were diagnosed according to the guidelines for the diagnosis and treatment of β-TM (2017) (28), developed by the Blood Group of the Chinese Medical Association and Pediatrics Branch. The inclusion criteria included: (1) age ≥ 18 years old; (2) genotype of β- thalassemia; (3) severity of thalassemia as major; (4) understanding the content of the questionnaire; (5) questionnaires were completed without missing data and had passed two rounds of quality control.

### 2.3. Data collection

A standardized questionnaire including patient's characteristics, disease status (e.g., co-morbidities, pre-transfusion hemoglobin level, transfusion burden, interruption of transfusion treatment, and interruption of iron chelation treatment), social support, caregiver burden, and QoL, was designed to collect data.

Transfusion burden was assessed by the average number of red blood cell transfusion (RBCT) units per 12 weeks. Transfusion burden was divided into non-severe transfusion burden (0 to < 12 RBCT units) and severe transfusion burden (≥12 RBCT units) (29).

Social support was evaluated by the social support rating scale (Cronbach α = 0.724) (30). It contained a total of 10 items in three dimensions with three items for objective support, four items for subjective support, and three items for utilization of social support. The total score is the sum of the scores for each item, ranging from 0 to 40. The higher the score of each dimension and the total score, the higher the level of social support. The level of social support was considered satisfactory if the total score was > 30 points.

Caregiver burden was measured by the Zarit burden interview (Cronbach α = 0.921) (31). It is scored on a 5-point likert scale from 0 to 4 with 22 items; thus, the total score ranges from 0 to 88 with higher scores indicating a heavier caregiver burden. The caregiver burden was categorized as non-severe caregiver burden (< 40 points) and the severe caregiver (≥40 points).

QoL was measured using the Chinese version of the SF-36 (Cronbach α = 0.854) with the score ranges from 0 to 100 (32), which is considered as a standard measurement tool for assessing QoL used worldwide (18). The main outcome measure was HSU, which was estimated through SF-36 responses using the SF-6D algorithm with the Hong Kong value set (33, 34). The total score ranges from 0.32 to 1.00 (35), and higher scores indicate better health status. SF-6D includes six dimensions: physical functioning (PF), role limitation (RL), social functioning (SF), body pain (BP), mental health (MH), and vitality (VT), and the six dimensions are derived from the 11 entries of the SF-36 scale. Each dimension contains four to six answers, i.e., levels, representing different health stages, with level 1 being the best and level 6 being the worst. Thus, there are a total of 18,000 health states (35). The RL dimension has only four levels, while the SF, MH, and VT dimensions both have five levels.

### 2.4. Quality control

Three investigators were recruited from Shandong University for arranging to carry out a strict check of the questionnaire completion through one-on-one phone calls according to the quality control plan to ensure that there were no missing items, logical errors or irregularities in the completion.

During the first round of quality control, a total of 219 questionnaires were returned, of which, 37 were for children and 78 were not diagnosed with β-thalassemia. The number of passing the final first quality control was 104. During the second round of quality control, out of 104 questionnaires, 23 were diagnosed with intermediate thalassemia and six patients were lost. The number of passing the final quality control 75, all of which were enrolled in the study sample.

### 2.5. Statistical analysis

We described the frequencies of options of each item of SF-6D answered by participants. The continuous variables were expressed as mean [Standard Deviation (SD)], and the categorical variables were as frequency (%). Then, the effect of each variable on the HSU was evaluated by univariable analyses. In combination with evidence from previous studies (17, 36), we included gender, age, nationality, province, level of education, student or not, comorbidities, interruption of transfusion treatment, interruption of iron chelation treatment, household catastrophic health expenditure, transfusion burden, social support, caregiver burden and pre-transfusion hemoglobin level of adult patients with β-TM as the predictors of OoL in adult patients with β-TM, and we performed the multiple linear regression model with ordinary least square (OLS) in order to control for the effect of parameters on HSU. The statistical significance level was indicated as *P*-value < 0.05, and the statistical borderline significance level as *P*-value < 0.1 (37). All statistical analyses were performed using SPSS statistics (SPSS version 24.0, Inc., Chicago, IL, USA).

### 2.6. Ethics approval

This study was approved by the Medical Ethics Committee of the Center for Health Management and Policy Research, Shandong University (No. ECSHCMSDU20211101). All participants were voluntary and anonymized, and they signed an online informed consent form before collecting data.

## 3. Results

### 3.1. Sample characteristics

A total of 75 adult patients were included after two rounds of quality control follow-ups in this study. The average age and pre-transfusion hemoglobin level were 24.7 years and 76.27 g/L, respectively. About half of the adult patients were males, or had an education level of high school and below. About 40% of adult patients interrupted transfusion therapy (44.0%), interrupted iron removal therapy (38.7%). The majority of them were diagnosed with co-morbidity (66.7%) and incurred catastrophic health expenditures (77.3%). In addition, it was reported that 65.3% of adult patients were student and 16% were the minorities. Approximately 60% were from Guangdong Province, 30.7% from Guangxi Zhuang Autonomous Region, 2.7% from Hunan province and Fujian province, respectively, and 1.3% from Jiangsu province, Jiangxi province, and Xinjiang Uyghur Autonomous Region, respectively (Table 1).

**Table 1:** Characteristics of adult patients with β-thalassemia major (*n* = 75).

**Variables**	***N* (%)**
Gender (male)	38 (50.7)
Age, years	
Mean (SD)	24.7 (5.6)
≤ 22 years	38 (50.7)
Nationality (minority)	12 (16.0)
Province	
Guangdong Province	45 (60.0)
Guangxi Zhuang Autonomous Region	23 (30.7)
Hunan Province	2 (2.7)
Fujian Province	2 (2.7)
Jiangsu Province	1 (1.3)
Jiangxi Province	1 (1.3)
Xinjiang Uygur Autonomous Region	1 (1.3)
Level of education ( ≤ high school and below)	37 (49.3)
Student or not (yes)	49 (65.3)
Co-morbidity (yes)	50 (66.7)
Transfusion burden (severe)	49 (65.3)
Interruption of transfusion treatment (yes)	33 (44.0)
Interruption of iron chelation treatment (yes)	29 (38.7)
Pre-transfusion hemoglobin level, g/L, mean (SD)	76.27 (12.3)
Household catastrophic health expenditure (yes)	58 (77.3)
Social support score	
Mean (SD)	30.6 (5.5)
≤ 30 scores	38 (50.7)
Caregiver burden score	
Mean (SD)	38.2 (16.7)
Severe	31 (41.3)

SD, standard deviation.

The average scores social support and caregiver burden were 30.6 and 38.2, respectively. Severe caregiver burden was present in a total of 41.3% of patient caregivers. Nearly half of the patients reported unsatisfactory levels of social support (Table 1).

### 3.2. QoL

The mean score for the physical component summary (PCS) was 47.6 ± 11.3, with the lowest and highest scores in the BP (30.1 ± 23.2) and PF (66.6 ± 19.2) dimensions, respectively. In addition, the mean mental component summary (MCS) score was 52.9 ± 14.2, with the lowest score with 44.0 was obtained for SF, and the highest score with 58.1 was for MH (Supplementary Table 1). 5.3, 26.7, 12.0, 25.3, 6.7, and 1.3% of participants chose the highest level of health status in the six dimensions of the SF-6D (PF, RL, SF, BP, MH, VT). Meanwhile, 40% of participants chose the worst level of health status in the RL dimension. In both of the SF and BP dimensions, only 1.3% of participants chose the worst level of health status (Table 2).

**Table 2:** Frequencies of each level in six dimensions of SF-6D for adult patients with β-thalassemia major (*n* = 75).

**Dimension**	**PF (%)**	**RL (%)**	**SF (%)**	**BP (%)**	**MH (%)**	**VT (%)**
Level 1	5.3	26.7	12.0	25.3	6.7	1.3
Level 2	20.0	28.0	30.7	2.7	40.0	16.0
Level 3		5.3	40.0	48.0	40.0	38.7
Level 4	62.7	40.0	16.0	17.3	10.7	34.7
Level 5			1.3	5.3	2.7	9.3
Level 6	12.0			1.3		

PF, physical function; RL, role limitation; SF, social function; BP, body pain; MH, mental health; VT, vitality.

Level 1–6: The answer under each dimension, represents different health stages, with level 1 being the best and level 6 being the worst.

### 3.3. Univariate analysis and correlation of HSU

The mean [95% confidence interval (CI)] of SF-6D HSU values was 0.598 (95% CI: 0.531–0.644; **Table 4**). In the univariate analyses, whether interruption of iron chelation treatment or not was reported a statistical significant association with HSU (P=0.038), and whether diagnosed with co-morbidity or not was reported a statistical borderline significant association with HSU (*P* = 0.0996). There were no significant differences in other parameters on HSU; however, we found that male, younger, worker, adult patients without severe transfusion burden, interruption of transfusion treatment, interruption of iron chelation treatment, household catastrophic health expenditure, and severe caregiver burden score, and those with higher education and higher social support, were significantly associated with higher HSU (Table 3).

**Table 3:** Differences in health state utility scores across subgroups for adult patients with β-thalassemia major (*n* = 75).

**Variables**		**Mean (95% CI)**	**SD**	***P*-value**
HUS	0.598 (0.531–0.644)	0.112	
Gender	Male	0.617 (0.578–0.657)	0.120	0.152
	Female	0.577 (0.543–0.611)	0.101	
Age (years)	≤22	0.618 (0.574–0.662)	0.134	0.225
	>22	0.576 (0.549–0.603)	0.081	
Nationality	Han nationality	0.597 (0.572–0.622)	0.099	0.544
	Minority	0.599 (0.490–0.707)	0.171	
Province	Guangdong	0.595 (0.563–0.627)	0.106	0.986
	Guangxi	0.604 (0.552–0.657)	0.121	
	Other	0.588 (0.464–0.712)	0.134	
Level of education	≤High school	0.579 (0.546–0.611)	0.099	0.266
	>High school	0.615 (0.576–0.656)	0.123	
Student or not	Yes	0.600 (0.567–0.634)	0.117	0.811
	No	0.592 (0.550–0.634)	0.104	
Co–morbidity	Yes	0.579 (0.553–0.605)	0.090	**0.0996**
	No	0.635 (0.576–0.693)	0.142	
Transfusion burden	Non-severe	0.605 (0.568–0.650)	0.104	0.544
	Severe	0.594 (0.562–0.625)	0.117	
Interruption of transfusion treatment	Yes	0.591 (0.546–0.635)	0.126	0.399
	No	0.603 (0.571–0.635)	0.102	
Interruption of iron chelation treatment	Yes	0.554(0.531–0.578)	0.061	**0.038**
	No	0.625(0.587–0.662)	0.128	
Household catastrophic health expenditure	Yes	0.595 (0.566–0.623)	0.109	0.535
	No	0.606 (0.541–0.670)	0.125	
Social support score	≤30	0.578 (0.552–0.603)	0.078	0.149
	>30	0.618 (0.572–0.664)	0.135	
Caregiver burden score	Non-severe	0.608 (0.572–0.643)	0.115	0.417
	Severe	0.583 (0.544–0.623)	0.108	

HUS, health utility state; CI, confidence interval; SD, standard deviation.

Other provinces included Fujian Province, Jiangsu Province, Jiangxi Province, Hunan Province, and Xinjiang Uygur Autonomous Region. The bold values indicated the *P*-value < 0.1.

Co-morbidity, pre-transfusion hemoglobin level and interruption of iron chelation treatment were significantly related to HSU (Supplementary Table 2).

### 3.4. Multivariate analysis of HSU

In the OLS regression model, the significant factors associated with HSU included co-morbidity, pre-transfusion hemoglobin level, and social support score. The most influential variable was co-morbidity. Patients with co-morbidities had significantly lower HSU values compared with those without co-morbidities. The pre-transfusion hemoglobin level and social support score were significantly positively associated with HSU values (Table 4).

**Table 4:** Multivariable regression models for factors associated with SF-6D utility scores for adult patients with β-thalassemia major (*n* = 75).

**Variables**	**B**	**SE**	**β**	***P*-value**
(Constant)	0.327	0.159		0.044
Female (ref: male)	−0.023	0.027	−0.103	0.389
Age (years)	0.0001	0.003	−0.007	0.957
Province (ref: Guangdong)				
Guangxi Zhuang Autonomous Region	0.042	0.032	0.175	0.193
Other	0.019	0.049	0.049	0.700
Student (ref: no)	0.032	0.032	0.126	0.319
Co-morbidity (ref: no)	−0.061	0.029	−0.257	**0.042**
Transfusion burden (ref: non-severe)	−0.020	0.029	−0.084	0.500
Interruption of transfusion treatment (ref: no)	−0.003	0.028	−0.014	0.911
Interruption of iron chelation treatment (ref: no)	0.041	0.029	0.177	0.168
Pre-transfusion hemoglobin level (g/L)	0.002	0.001	0.252	**0.047**
Social support score	0.005	0.002	0.226	**0.068**
Caregiver burden score	−0.001	0.001	−0.108	0.374

SE, standard error.

Other provinces included Fujian Province, Jiangsu Province, Jiangxi Province, Hunan Province, and Xinjiang Uygur Autonomous Region. The bold values indicated the *P*-value < 0.1.

## 4. Discussion

To our knowledge, this study is the first study measuring HSU values and identifying the influencing factors for adult patients with β-TM in mainland China. The QoL and HSU among adult patients with β-TM was poor. Diagnosis with comorbidity was significantly negatively associated with HSU values, and pre-transfusion hemoglobin levels and social support were significantly positively associated with HSU values. It would assist policymakers in the decision-making process related to disease management in the thalassemia patients, especially considering β-TM has become a public health problem in areas with high prevalence in China (28).

Soheir et al. found the presence of chronic pain was closely associated with lower QoL outcomes of thalassaemia patients (38). This is consistent with our finding that the mean score for PCS was lower than MCS and the BP dimension had the lowest score among all domains. BP in patients with β-TM was strongly associated with lower PCS scores, limited their daily activity and affected SF (38), which could be attributed to low hemoglobin concentration, low bone mass, and/ or iron overload (39). Physical health effects of thalassemia may lead to physical malformation, growth retardation, delayed puberty, poor self-image, and patients with serious complications such as heart failure, cardiac arrhythmia and liver disease (39). The absence of certain blood groups or the shortage of blood in some situations had affected the blood-dependent life of thalassemia patients (8). Blood reactions, alloimmunization and antibody formation in the blood, as well as viral infections through infected blood had threatened the physical health of patients (8). Thus, Francesca et al. suggested that compliance with treatment contributes to the health of patients, not only physically, but also psychologically (40).

In addition, although the result of the highest MCS score was in line with the finding of Bazi et al. (19), it is essential to emphasize the importance of mental health in patients with β-TM. The minimum value of SF observed in the MCS was contrary to the published meta-analysis of 26 studies (18). A large quantity of evidence confirmed that psychological problems were the main problem in thalassemia patients (41, 42). It was found that Greek thalassemia patients suffered more psychological problems than physical functioning (43). One study reported that thalassemia patients had the most mental health and role emotional problems, had no life satisfaction and suffered from depression and anxiety (44). Khani et al. reported that 64.9% of patients were candidates for psychiatric medication (44). Uncertainty about the future of the disease and treatment, on the one hand, and worries about the high cost of treatment, on the other hand, worries about playing a social role and the future of the family, can cause psychological stress for thalassemia sufferers and endanger their mental health (8).

We found that SF-6D HSU values in our study (0.598) was obviously lower than the HSU value of 0.95 for EQ-5D in the all-age Chinese general population (45). In addition, it was reported that thalassemia patients were 0.893 HSU values measured by EQ-5D in Malaysia (1). In another study, Iranian patients with transfusion-dependent thalassemia (TDT) using iron chelation therapy had HSU values ranging from 0.81 to 0.86 as measured by the time-weighted value set (46), which were higher than the result in our study. Differences in HSU values may be related to the different instruments used to measure QoL, the different characteristics of study population in different countries, and the different treatments received by patients.

The EQ-5D focuses primarily on the physical domain of measuring QoL with four dimensions reflecting physical aspects and one dimension for mental health. β-TM has a direct impact on patients' physical health, and other complications from frequently administered blood transfusions and iron chelation therapy increase the psychological burden. SF-6D provides a more comprehensive physical, psychological, social, and emotional reflection of QoL. Therefore, it is an appropriate tool to measure the impact of β-TM on patients' health. Results from other studies in the UK and the US indicated that the SF-6D was more discriminative than the EQ-5D-5L (47). Brazier and colleagues developed the SF-6D scale based on the SF-36 scale (48) and extracted utilities from the SF-36 by using the utility weights of the UK population (35). The SF-6D has subsequently seen widespread use in many countries. Lam conducted a pre-test in Hong Kong, China, in 2008 (34) and developed a more sophisticated SF-6D utility value score system in 2011 (33).

We found that respondents tended to report the most severe problems on the RL dimension. Although no performance of the SF-6D in the population of thalassemia was reported in previous studies, research on other patients showed similar findings. For example, a UK study indicated that 38.4% of patients with seven different disorders reported extreme problems with RL (49). Longworth et al. found that 42.4% of liver transplant patients reported the most severe problems on the RL dimension (47). Therefore, the performance of SF-6D in populations from different countries or regions requires further examinations.

This study indicated that co-morbidities among adult patients with β-TM were associated with lower HSU values. There was a similar result reporting that co-morbidities had a significantly negative effect on HSU scores in an Iranian study (46). In addition, several studies supported the association between adherence to iron removal therapy and QoL (40, 50), which highlighted the importance of regular iron removal therapy (50). However, it was reported that patients with β-TM were associated with poor adherence of transfusion treatment and iron removal treatment, which was similar to the result in our study (40).

This study found that lower pre-transfusion hemoglobin levels negatively affected QoL, which is consistent with the result of previous studies (51, 52). Several studies indicated that patients with more severe anemia tended to have a worse QoL (51, 53). One study using PedsQL 4.0 Generic Core Scale reported that patients with pre-transfusion hemoglobin levels >90 g/L had significantly higher QoL scores than those with the level of 70–90 g/L or < 70 g/L (51). One study conducted in Omani children found that those with higher pre-transfusion hemoglobin levels were associated with better QoL scores (53). Another study from Thailand showed that most patients with β-TM died from anemia, suggesting that a high proportion of patients received inadequate transfusion (54). Patients with β-TM would be exposed to the progressive effects of chronic anemia if they did not receive a timely transfusion or transplantation, and die within a few months of diagnosis (55). Therefore, regular blood transfusions and chelation therapy could improve survival rate and QoL in patients with β-TM (5, 56) and transform a previously fatal disease with death into a chronic disease compatible with prolonged survival. However, the poor compliance to blood transfusion and iron chelation were reported in this study, which was consistent with the finding of another Egyptian study (57). If without regular transfusion and iron chelation therapy, the morbidity and mortality of thalassemia would be similar to or higher than leukemia in the future (55).

This study showed that adult patients with β-TM had low social support scores, which is in alignment with many studies (23, 58, 59). Social support would help relieve and catharsis of negative psychological emotions, allowing patients and families to reduce anxiety and fear of illness (60). As compared to other patients, this group frequently exhibits maladaptive coping strategies and highly anxious psychosocial dysfunction due to the distress from the illness itself and the need for iron chelation (8). The lack of appropriate social status for them and poor acceptance of thalassemia patients in society have made these patients feel frustrated, fearful and uncertain about the future (8). Society generally did not accept these patients appropriately. Such inequalities with their healthy peers added to the mental abnormalities of thalassemia patients (44). Khani et al. found that 87 and 35% of thalassemia patients were usually single, having marital and occupational problems, respectively (44), which might be one of the leading causes of anxiety and depression. Kumar et al. study in Singapore also showed that thalassemia major patients feared disclosure of their disease that was due to the concern about the stigma of the inability to marry and work, as well as the fear of social and occupational discrimination (61). Access to social support and health care indicated better QoL in a combined intercontinental study designed to measure QoL and care for thalassemia patients in Lebanon, Canada and Iran (58). These findings emphasize the need for social support for thalassemia major patients and the need for social destigmatization.

In addition, nearly 40.7% of the caregivers in this study had moderate to severe caregiving burdens, which was consistent with another study (62). A cross-sectional study from Malaysia indicated that nearly half of the caregivers experienced psychiatric problems of anxiety or depression (42). This chronic and unending illness is a source of stress for the parents and the rest of the family, and also places a clinical burden on the whole family which includes psychological and social consequences (62). Fully mobilizing and utilizing other social support resources and those of relatives aimed at reducing the burden of the parents, and strengthening their coping strategies for better integration in daily life, is therefore essential for adult patients and caregivers with β-TM.

Concordant with the findings from a previous study, gender was not related to QoL (63). However, we observed the QoL to be significantly higher in males than in females between gender subgroups, as in previous studies (19, 41). Contrary to what Floris et al. reported, they thought females were better at adhering to treatment plans and lifestyles (40). A higher educational level was not found to be a predictor of QoL, while HSU values were significantly better in patients with higher education levels. In Egypt and Sardinia, significantly higher QoL scores were reported for patients with higher education levels (40, 59). The possible explanation is that patients with higher education levels were more likely to have a better understanding of the disease and superior adherence to treatment. We did not find a significant association between patient's age and QoL, which was inconsistent with the previous studies (38, 46, 51). Seyedifar et al. indicated that age was also an important factor affecting QoL in thalassemia patients, and increasing age was accompanied by increasing anxiety and depression, as well as an increasing prevalence of diabetes and hepatitis (46). Dahlui et al. demonstrated that age growth with a decrease in HSU (63). The studies of Teawtrakul et al. (21) and Haghpanah et al. (41) found that older age was correlated with increased comorbidities and reduced QOL. However, in our study, we only included adult patients, and most patients were in their early adulthood, so it was difficult to observe differences in QoL across age subgroups.

This study has a few limitations. First, as a cross-sectional design, the association between QoL and factors cannot be interpreted as causal. Second, the sample size was relatively small, and it might limit the generalization of the results. There were some possible reasons: (1) the lack of epidemiological distribution information nationwide, which made it difficult to conduct effective sample estimation and field survey; (2) under the effects of rather scattered patient residence, COVID-19, patients' reluctance to participate in face-to-face surveys and other factors, it was difficult to conduct onsite surveys, we had to choose online survey by snowball sampling through referrals from physicians and patients. However, as adult patients were a fairly small group, the sample in this study was still representative to reflect their situation. Third, this study was an online survey, and might be susceptible to information bias; however, we conducted two-rounds quality control follow-ups to mitigate this effect. Further empirical prospective studies with large sample size are needed in the future.

## 5. Conclusion

Adult patients with β-TM were associated with lower QoL and HSU in mainland China. Comorbidity, pre-transfusion hemoglobin level, and social support were independent risk factors for the HSU value among adult patients with β-TM. It highlights the importance of adherence to transfusion treatment and iron chelation treatment. The findings are usable in the disease management decision-making process and will be useful for researchers to employ in future clinical and health economic studies, including decision analysis and cost-effectiveness analysis.

## Data availability statement

The original contributions presented in the study are included in the article/Supplementary material, further inquiries can be directed to the corresponding authors.

## Ethics statement

This study was approved by the Medical Ethics Committee of the Center for Health Management and Policy Research, Shandong University. All patients and caregivers were voluntary and signed an online informed consent form before collecting data. The patients/participants provided their written informed consent to participate in this study.

## Author contributions

RZ participated in the conception, design of this study, data collection, data analysis, interpretation of data, and drafted and revised the manuscript. JM, RZ, JX, and BL performed the data analysis, interpretation of data, and drafted and revised the manuscript. CC assisted in the data collection. XZ participated in the conception, design of the study, data collection, interpretation of data, and drafted and revised the manuscript. XS participated in the conception, design of the study, and helped in the revising the manuscript. All authors contributed to the article and approved the submitted version.
