# Supplementary material for: Predictors of health state utility values using SF-6D for Chinese adult patients with β-thalassemia major
Source: Front Public Health. 2023 Jan 19;10:1072866. doi: 10.3389/fpubh.2022.1072866 (PMC9892772; doi:10.3389/fpubh.2022.1072866)
Supplement: Supplementary file 1 [file Table_1.DOCX]

Table S1. Overall QoL scores of adult patients with β-thalassemia major (n = 75).

| SF-36 Dimension | Mean ± SD |
| --- | --- |
| Overall Score | 50.2 ± 10.7 |
| Physical Component Summary (PCS) | 47.6 ± 11.3 |
| Physical Functioning (PF) | 66.6 ± 19.2 |
| Role Physical (RP) | 35.0 ± 41.1 |
| Body Pain (BP) | 30.1 ± 23.2 |
| General Health (GH) | 58.6 ± 10.5 |
| Mental Component Summary (MCS) | 52.9 ± 14.2 |
| Vitality (VT) | 56.9 ± 12.3 |
| Social Functioning (SF) | 44.0 ± 12.2 |
| Role Emotional (RE) | 52.4 ± 47.5 |
| Mental Health (MH) | 58.1 ± 11.6 |

QoL: quality of life; SD: standard deviation.

Table S2. Pearson correlation of selected features and HSU (n = 75).

| Variables | P-value |  |
| --- | --- | --- |
|  | HSU | |
| Gender | 0.1204 | |
| Age, years | 0.1052 | |
| Nationality | 0.9610 | |
| Province | 0.9579 | |
| Level of education | 0.1539 | |
| Student or not | 0.7563 | |
| Co-morbidity | 0.0420 | |
| Transfusion burden | 0.3584 | |
| Interruption of transfusion treatment | 0.6342 | |
| Interruption of iron chelation treatment | 0.0073 | |
| Pretransfusion hemoglobin level, g/L | 0.0217 | |
| Household catastrophic health expenditure | 0.7388 | |
| Social support score | 0.1191 | |
| Caregiver burden score | 0.2483 | |

QoL: quality of life; HSU: health state utility.
